# Analysis of the Clinical Characteristics of Tuberculosis Patients based on Multi-Constrained Computed Tomography (CT) Image Segmentation Algorithm

**DOI:** 10.12669/pjms.37.6-WIT.4795

**Published:** 2021

**Authors:** Feng Zhu, Bo Zhang

**Affiliations:** 1 Feng Zhu, Attending Doctor Department of Respiratory and Critical Care Medicine, The Second Clinical Medical College, Yangtze University, Jingzhou Central Hospital, Jingzhou 434000, China; 2 Bo Zhang, Attending Doctor, Radiological Department, The Second Clinical Medical College, Yangtze University, Jingzhou Central Hospital, Jingzhou 434000, China

**Keywords:** pulmonary tuberculosis, lung cancer, diagnosis, clinical manifestations, image segmentation algorithm, CT imaging features

## Abstract

**Objective:**

We used U-shaped convolutional neural network (U_Net) multi-constraint image segmentation method to compare the diagnosis and imaging characteristics of tuberculosis and tuberculosis with lung cancer patients with Computed Tomography (CT).

**Methods:**

We selected 160 patients with tuberculosis from the severity scoring (SVR) task is provided by ImageCLEF Tuberculosis 2019. According to the type of diagnosed disease, they were divided into tuberculosis combined with lung cancer group and others group, all patients were given chest CT scan, and the clinical manifestations, CT characteristics, and initial suspected diagnosis and missed diagnosis of different tumor diameters were observed and compared between the two groups. The research continued for a year in the office, mainly relying on a computer with GPU to carry out graphics analysis.

**Results:**

There were more patients with hemoptysis and hoarseness in pulmonary tuberculosis combined with lung cancer group than in the pulmonary others group (P<0.05), and the other symptoms were not significantly different (P>0.05). Tuberculosis combined with lung cancer group had fewer signs of calcification, streak shadow, speckle shadow, and cavitation than others group; however, tuberculosis combined with lung cancer group had more patients with mass shadow, lobular sign, spines sign, burr sign and vacuole sign than others group.

**Conclusion:**

The symptoms of hemoptysis and hoarseness in pulmonary tuberculosis patients need to consider whether the disease has progressed and the possibility of lung cancer lesions. CT imaging of pulmonary tuberculosis patients with lung cancer usually shows mass shadows, lobular signs, spines signs, burr signs, and vacuoles signs. It can be used as the basis for its diagnosis. Simultaneously, the U-Net-based segmentation method can effectively segment the lung parenchymal region, and the algorithm is better than traditional algorithms.

## INTRODUCTION

Pulmonary tuberculosis is a chronic infectious disease caused by Mycobacterium tuberculosis with a high incidence rate. It is a significant public and social problem worldwide. Both tuberculosis and lung cancer have clinical symptoms such as cough, sputum, hemoptysis, and their clinical features overlap. It is difficult to distinguish only from clinical symptoms and signs. Some researchers have pointed out that the comorbidity of tuberculosis and lung cancer is more familiar with increased clinical incidence of tuberculosis or lung cancer. Computer-aided diagnosis (CAD) can help doctors automatically detect and analyze diseases.

To implement a CAD system, it is necessary to segment human organs and structures from computer tomography (CT) images. The lung CT scan image is mainly composed of the lung parenchyma, pulmonary blood vessels, and bronchi. Segmenting the lung parenchymal area from the CT image can improve the CAD system’s efficiency and reduce misdiagnosis. Therefore, lung images’ segmentation effect can affect the accuracy of the entire lung CAD system, which is a critical step in diagnosing lung diseases. At this stage, lung CT imaging is still the primary method for diagnosing lung cancer in China. However, when tuberculosis and lung cancer coexist, lung cancer diagnosis and treatment are easily delayed due to similar symptoms. Therefore, the lung CT imaging of the two is identified. The characteristics are of great significance for guiding the clinical diagnosis of tuberculosis with lung cancer.

Investigators at home and abroad have proposed many lung CT image segmentation algorithms based on the apparent differences between the lung parenchyma and surrounding tissues.^1^ In response to this problem, this study selected tuberculosis combined with lung cancer group and tuberculosis patients treated in our hospital to further compare the CT diagnosis and imaging characteristics of the two patients. At the same time, we used a U-shaped convolutional neural network (U-Net) to segment the lung CT image.

## METHODS

The dataset for the severity scoring (SVR) task is provided by ImageCLEF Tuberculosis 2019.^2^ The dataset consists of a total 160 chest CT scans of Tuberculosis patients in addition with clinically relevant metadata (part of the data is filtered, the number of original data sets is 335). The selected metadata includes the following binary measures: disability, relapse, symptoms of TB, comorbidity, bacillary, drug resistance, higher education, ex-prisoner, alcoholic, smoking. The 3D CT images which were provided have a slice size of 512×512 pixels and a number of slices varying from 50 to 400. All the CT images are stored in NIFTI file format.

The segmentation algorithms for lung CT images are mainly divided into threshold-based methods, boundary-based methods, and specific theory-based methods.^3^ The lungs are filled with a lot of air, and the lungs are a black area in the CT scan image. Simultaneously, the contrast between the lungs and surrounding tissues is evident, making many researchers try to find an optimal threshold to separate the lung area, including the gray threshold method, histogram threshold method, and 3D threshold method. However, due to image brightness change, these segmentation effects are not ideal.

The boundary-based method uses edge detection filters or wavelet transform to detect discontinuous boundary pixels and then connect them to form a complete boundary. Some authors use first-order Gaussian filters in various directions to detect the lungs’ boundary contours and then use LoG operators of different scales for boundary tracking to find the contours of the lungs. Methods based on specific theories introduce various mathematical theories^4^ into image segmentation, such as segmentation methods based on fuzzy theory, segmentation methods based on mathematical morphology, and segmentation methods based on deformation models.^5^

**Fig.1:**
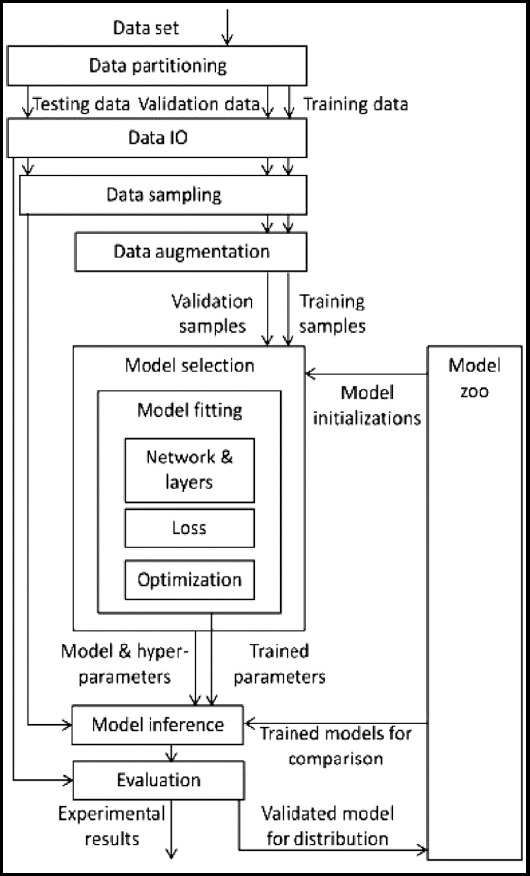
Overall flow chart.

This article uses an individual transformation to enhance the CT image’s details and uses a parallel U-Net network to improve the image segmentation effect. The process of the method proposed in the article is shown in Fig.1.

When selecting image processing methods, we consider processing efficiency. The more image processing methods selected, the more detailed information can be obtained from CT images, but the processing efficiency will also be reduced. Therefore, we selected two processing methods, Gaussian filtering, and Laplacian. The study selected linear smoothing filter-Gaussian filter to eliminate Gaussian noise and remove noise points; chooses the Laplacian Klaplace shown in formula (1) to sharpen the microvascular structure make it easy to segment. This article separates the two image processing methods to avoid smoothing out the delicate blood vessel structure.


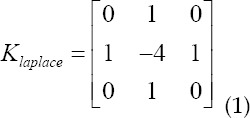


### U-Net segmentation:

This paper is based on the U-Net network proposed in the literature. 6

The U-Net requires fewer training samples, is fast, and has a better effect than sliding convolutional networks.^7^ Use the lung CT image and its corresponding segmentation map to train the network. The network uses the energy function to calculate the soft-max value of each pixel.


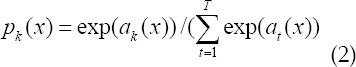


Among them, *^a_k_(x)^* represents the activation value of the feature channel k at the pixel position x, k represents the number of categories and *^p_k_(x)^* is the approximate maximum function. In this paper, the binary cross-entropy of each pixel is used as the target to train the model, and the gradient descent method (SGD) is used to train the loss function to make it converge to a minimum. The update strategy is:


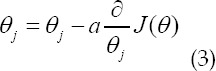


Among them, α is the learning rate, and a fixed value of 0.003 is used for learning.

### Linear fusion:

As shown in Fig.1, this paper segmented the preprocessed image in parallel to obtain multiple segmentation results, so it is necessary to fuse each network’s segmentation results to obtain the final lung segmentation image. Simultaneously, considering the deviation of different segmentation results, different weights are used for different segmentation results.^8^ This paper uses linear regression to fuse to obtain the final segmentation result.

SPSS software was used for analysis. The clinical manifestations, CT characteristics, initial suspected diagnosis, and missed diagnosis of the two groups were all counted, expressed by frequency, and compared by the chi-square test. P<0.05 indicates that the difference is significant.^9^

## RESULTS

There were more patients with hemoptysis and hoarseness in pulmonary tuberculosis combined with lung cancer group than in the pulmonary tuberculosis group. The difference was significant (P<0.05), and the other symptoms were not significantly different (P>0.05), Table-I.

**Table-I:** Comparison of CT characteristics of two groups of patients (cases).

*Group*	*Number of cases*	*Calcification*	*Strip shadow*	*Flake shadow*	*Speckle*	*Clump shadow*
Tuberculosis with lung cancer group	80	4	46	24	45	71
Tuberculosis group	80	13	70	17	60	10
*χ*2		5.331	18.056	1.607	6.234	93.04
P		0.021	<0.001	0.205	0.013	<0.001

*Group*	*Number of cases*	*Void sign*	*Leaf sign*	*Spines sign*	*Glitch sign*	*Vacuole sign*

Tuberculosis with lung cancer group	80	9	47	35	37	13
Tuberculosis group	80	26	3	6	10	2
*χ*2		10.569	56.32	25.579	21.962	8.901
P		0.001	<0.001	<0.001	<0.001	0.003

Tuberculosis combined with lung cancer group had fewer signs of calcification, streak shadow, speckle shadow, and cavitation than tuberculosis group; however, tuberculosis combined with lung cancer group had more patients with mass shadow, lobular sign, spines sign, burr sign, and vacuole sign than tuberculosis group.^10^ And the difference is significant (P<0.05). Table-I.

**Table-II:** The accuracy of lung segmentation for different images.

	Fig.1	Fig.2	Fig.3	Fig.4
JS	0.991	0.978	0.983	0.997

At the same time, this paper also uses the Jaccard Similarity (JS) indicator to quantitatively evaluate the proposed segmentation algorithm. The following formula shows the definition of JS.


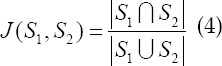


Among them, S1 is the reference manual annotation result, and S2 is the segmentation result obtained by the algorithm in this paper. As shown in Table-II, it is the final accuracy rate of four randomly selected CT images. It can be seen that the accuracy of this algorithm has reached 99%, and it can segment the lung area better.^11^

## DISCUSSION

Both pulmonary tuberculosis and lung cancer are common pulmonary diseases with a relatively high prevalence. The pathogenesis of pulmonary tuberculosis is a tuberculosis infection, which is mainly transmitted through the respiratory tract. Lung cancer mainly occurs in the mucosal epithelium of the air duct. It is one of the malignant tumors with a high mortality rate. Although the two have different pathogenesis, some scholars have pointed out that many epidemiological studies believe that the decline of the immunity of lung cancer patients can easily lead to tuberculosis infection and then produce tuberculosis.^12^ The misdiagnosis rate of tuberculosis is a little high.^13^ Tuberculosis has a particular promotion effect on lung cancer; namely, one of them may create conditions for the other to happen. Besides, because they both occur in the lungs, they have more similar clinical features and are difficult to distinguish, which increases the difficulty of clinical diagnosis. Therefore, it has particular clinical value to explore how to distinguish the two by clinical symptoms.^14^ This study showed that more patients in tuberculosis combined with lung cancer group had hemoptysis and hoarseness than the tuberculosis group, and there was no significant difference in other symptoms. It is suggested that when pulmonary tuberculosis patients have severe hemoptysis, bloody pleural effusion, hoarseness, etc., it is necessary to consider whether the disease has progressed and the possibility of lung cancer lesions. Relevant studies also show that different types of patient groups have greater differences in compliance with treatment medications.^15^

Due to the lack of specificity of clinical symptoms, lung imaging is the primary method for diagnosing tuberculosis with lung cancer and uncomplicated tuberculosis.^16^ This study’s chi-square test results showed that tuberculosis combined with lung cancer group had fewer calcifications, streak shadows, speckle shadows, and cavitation signs than the pulmonary tuberculosis group.

Consolidated cancer tissues can be mixed with normal gas-filled alveoli, coupled with the uneven growth of cancer cells, causing signs of lobes, burrs, spines, and other signs on the edges of the nodules. Therefore, more pulmonary tuberculosis patients combined with the lung cancer group had the mass shadow, lobular sign, spines sign, burr sign, and vacuole sign than the pulmonary tuberculosis group. The patchy shadow is mainly the CT imaging feature of the early exudative lesions of pulmonary tuberculosis. Both have tuberculosis infection, so there is no significant difference between the two groups in patients with patchy shadow.

In patients with pulmonary tuberculosis, chronic stimulation of tuberculosis lesions can promote the necrosis and shedding of alveolar epithelial cells in the lesions, and adjacent bronchioles. Coupled with tuberculosis infection, it can cause tuberculosis calcification, scar tissue, tuberculosis nodules, etc. to appear in the bronchus. Local lymph and blood in the lung tissues cause local deposition of carcinogens and gradually progress to lung cancer. When tuberculosis and lung cancer overlap, imaging diagnosticians are easily guided by the idea of monism. For different imaging manifestations of the same patient, one disease manifestation can be used to explain the same disease diagnosis as much as possible, ignoring the existence of lung cancer and making a partial generalization. From the wrong interpretation of a diagnosis, misdiagnosis appear. Therefore, in clinical diagnosis, we should correctly understand the CT imaging features of pulmonary tuberculosis and lung cancer and make the early diagnosis for secondary lung cancer patients.

## CONCLUSIONS

In summary, hemoptysis and hoarseness in patients with tuberculosis need to be considered to develop the disease and the possibility of lung cancer. CT imaging of patients with tuberculosis and lung cancer is mostly manifested as mass shadows, lobular signs, spines, burrs, and voids. The bubble sign can be used as the basis for its diagnosis.

### Authors Contribution

**BZ** conceived, designed, did statistical analysis & editing of manuscript, is responsible for integrity of research.

**FZ** did data collection and manuscript writing.
